# A Descriptive Transcriptomic Resource of Head Tissues from Three Scarab Beetle Species at a Single Time Point Corresponding to Divergent Behavioral States

**DOI:** 10.3390/insects17080781

**Published:** 2026-07-28

**Authors:** Zhongjun Gong, Jing Zhang, Yun Duan, Huiling Li, Kebin Li, Weizheng Li, Yongsheng Yao, Yuqing Wu, Jin Miao

**Affiliations:** 1 Henan Key Laboratory of Agricultural Pest Monitoring and Control, Institute of Plant Protection, Henan Academy of Agricultural Sciences, Zhengzhou 450002, China; gongzj_2@hnagri.org.cn (Z.G.);; 2 Key Laboratory of Integrated Crop Pests Management on Crops in Southern Region of North China, Ministry of Agriculture and Rural Affairs, Institute of Plant Protection, Henan Academy of Agricultural Sciences, Zhengzhou 450002, China; 3 International Joint Research Laboratory for Crop Protection of Henan, Institute of Plant Protection, Henan Academy of Agricultural Sciences, Zhengzhou 450002, China; 4 State Key Laboratory for Biology of Plant Diseases and Insect Pests, Institute of Plant Protection, Chinese Academy of Agricultural Sciences, Yuanmingyuan West Road, Beijing 100193, China; 5 College of Plant Protection, Henan Agricultural University, Zhengzhou 450002, China; 6 College of Agriculture, Tarim University, Aral 843300, China

**Keywords:** *Holotrichia parallela*, *Holotrichia oblita*, *Anomala corpulenta*, transcriptome, head tissue

## Abstract

Two of the scarab beetle species examined here, *Holotrichia oblita* and *Anomala corpulenta*, show approximately 24 h intervals between locomotoractivity events; whereas, a third species, *H. parallela*, shows approximately 48 h intervals. We collected head tissues from all three species at a single time point, when the two 24 h rhythm species were activeand the 48 h rhythm species was not, and profiled their transcriptomes. Core clock genes showed comparable transcripts per million (TPM) valuesacross species at this time point. Expression differences were observed in genes involved in phototransduction (Gq alpha subunit isoforms) and mitochondrial energy metabolism (adenine nucleotide translocase isoforms). Additionally, 22 orthologs, including genes related to the ubiquitination cascade and 20S proteasome subunits, showed varying TPM values across species. Protein–protein interaction network analysis showed that many of these genes with varying TPM values cluster into conserved functional modules. This work is therefore presented solely as a descriptive transcriptome resource for these three beetle species at the collection time point.

## Simple Summary

Most insects follow a 24 h daily rhythm, but some species exhibit unique behavioral patterns. In this study, we investigated three closely relatedbeetle species that are major agricultural pests in Asia. *Holotrichia oblita* and *Anomala corpulenta* emerge at 24 h intervals; whereas, *Holotrichia parallela* emerges every 48 h, representing a rare biological phenomenon. We collected all three species at a single time point, when the 24 h species were emerging but the 48 h species was not, and profiled their head transcriptomes. Core clock genes showed comparable expression levels across species. Differences were observed in visionand protein degradation pathways. Due to the single time point, different behavioral states (active vs. non-active), and different host plants, expression differences cannot be attributed to a single factor. This study serves as a descriptive transcriptome resource at a single time point.

## 1. Introduction

Most animals exhibit daily biology rhythms in their lives, which are thought to be regarded as an adaptive mechanism in response to the 24 h light/dark cycle and the daily environmental cycles associated with the Earth’s rotation [1]. The rhythms are generated by an endogenous mechanism known as the circadian clock. Therefore, innate cyclical mechanisms aligned with environmental fluctuations enable organisms to predictively adapt their metabolic and behavioral states. Intriguingly, the large black chafer *Holotrichia parallela* (Coleoptera: Scarabaeidae), a major agricultural pest in East Asia, displays a unique circabidian rhythm in its locomotor activity patterns. Adult activity eventsoccurs every 2 days, a rhythm referred to circabidian rhythm, which is likely driven by the circadian clock [2]. Males are drawn to the pheromone during sunset, followed by mating activity. In the field, the emergence of the chafer on the ground takes place every two days [2,3,4,5,6]. This 48 h periodicity was also observed in the concentration of L-isoleucine methyl ester (LIME), the primary component of the *H. parallela* sex pheromone, showing approximately a 48 h periodicity [6]. The captures of *H. parallela* males using pheromone-baited traps also presented peaks every two days [6]. The sex pheromone receptor gene *HparOR14* exhibits a circabidian rhythm, with its transcript levels rising during the scotophase every two days, coinciding with the beetle’s mating behavior [7]. A recent study showed that *H. picea* also exhibits a circabidian rhythm [8]. All beetles that had their complete bilateral optic lobe (OL) removed lost their circabidian rhythm, suggesting that the OLs are crucial for the circabidian rhythm [9]. The observation of circadian-like activity patterns and day-switching behaviors in *H. parallela* and *H. picea* implies interesting questions about its evolutionary and physiological basis.

Scarab beetle larvae pose significant agricultural threats globally [10]. In China, three sympatric scarabaeoid species, two from the subfamily Melolonthinae (*H. parallela* and *H. oblita*) and one from Rutelinae (*Anomala corpulenta*), exhibit divergent adult locomotor activity rhythms despite larval niche overlap [11]. *H. parallela* displays a unique every-other-day locomotor activity rhythm, while its congener *H. oblita* and the more distantly related *A. corpulenta* both show activity daily post-sunset. This group of species includes a close relative (*H. oblita*) and a more distant relative (*A. corpulenta*) of *H. parallela*, providing a phylogenetic contrast in locomotor activity rhythms.

In this study, we conducted the head transcriptomes of three scarab beetle species with different locomotor activity rhythms. Samples were collected at 22:00 (nighttime) from all three species. Due to the single-time-point design and the different behavioral states at collection, we do not interpret the observed expression differences as causal evidence for the control of rhythm of locomotor activity. Instead, we describe the head transcriptome profiles of the three species and report correlations between circadian behavioral states and head gene expression at this specific collection time. Core clock genes showed comparable expression levels across species under this sampling condition. Differences in expression were observed in genes involved in phototransduction (Gq alpha subunit isoforms) and mitochondrial energy metabolism (adenine nucleotide translocase isoforms). Additionally, 22 orthologs showed differential expression across species. This study provides a descriptive transcriptomic resource for three scarab beetle species, providing a foundation for future studies with multi-time-point sampling.

## 2. Materials and Methods

### 2.1. Tissue Sampling

*A. corpulenta*, *H. oblita* and *H. parallela* were originally collected from the Henan Research and Development Center for Modern Agriculture (35°00′ N, 113°40′ E), Yuanyang County, Henan Province, China in July 2017. To minimize potential variations induced by age, we adopted an early-life sampling approach for the three beetle species, given that transcriptome profiles can differ across developmental stages among species. They were divided by sex and placed in boxes with soil and fresh leaves. The rearing took place at a temperature of 25 ± 1 °C, a relative humidity of 70%, and 14 L:10 D (6:00 h–20:00 h). During the recording period, *H. parallela* and *H. oblita* were provided with leaves of *Ulmus pumila* as their food source, while *A. corpulenta* was provided with leaves of *Ligustrum quihoui*. The beetles were continuously observed using infrared cameras to monitor their activity over an 8-day period under light–dark (LD) cycles. Behavioral monitoring confirms that three beetles had normal locomotor rhythms with peak activity concentrated around dusk. *H. parallela* showed a stable circabidian rhythm, with activity occurring every 48 h. Meanwhile, *A. corpulenta* and *H. oblita* exhibited 24 h circadian rhythm and appeared every day. There were no notable differences between males and females. For transcriptome sequencing, we collected heads from *H. parallela* during its inactive period, and from *A. corpulenta* and *H. oblita* during their active period, all at 22:00 h. For each species, we sampled three males and three females as independent biological replicates, giving 18 samples in total.

### 2.2. RNA Extraction and Sequencing

Transcriptome sequencing was performed on head tissues collected from adult individuals of both sexes, with three biological replicates per group. A total of 18 independent cDNA libraries were constructed. For RNA extraction, approximately 3 μg of total RNA was used for each biological sample. RNA purity was examined with the NanoPhotometer spectrophotometer (IMPLEN, Munich, Germany). RNA concentration was determined using the Qubit RNA Assay Kit on a Qubit 2.0 Fluorometer (Life Technologies, Carlsbad, CA, USA), while RNA integrity was evaluated using the RNA Nano 6000 Assay Kit on an Agilent Bioanalyzer 2100 system (Agilent Technologies, Santa Clara, CA, USA). The libraries were sequenced using the Illumina HiSeq 2500 platform (Illumina Inc., San Diego, CA, USA), generating paired-end reads. The RNA sequencing data have been submitted to NGDC (the National Genomics Data Center, Beijing Institute of Genomics, Chinese Academy of Sciences) with the accession number CRA011607 and CRA011598.

### 2.3. Transcriptome Assembly, Annotation, and Gene Analysis

For each species, we combined six cDNA libraries (three derived from male heads and three from female heads) before conducting de novo assembly to create a reference transcriptome for each species. Since no reference genome was available for any of the three scarab beetle species, de novo transcriptome assembly was performed for each species individually using Trinity (version r20140413p1) [12]. For functional annotation, the assembled unigenes were aligned to the protein sequences of *Tribolium castaneum* (a closely related beetle within Coleoptera) using BLASTX (v2.2.28+) (e-value < 1 × 10^−5^). Additional functional annotations were performed by searching against the following databases: Nr, Nt, Pfam, KOG, Swiss-Prot, KO, and GO. The simple sequence repeats (SSRs) were identified through the utilization of MISA software (version 1.0) [13]. Gene expression levels were estimated using RSEM v1.3.1 [14], with clean reads of each sample mapped to the Trinity assembled transcriptome. Unigene expression was calculated based on the transcripts per million (TPM) method [15]. Orthologous groups were identified using OrthoMCL v2.0.3 [16] based on BLASTP similarity searches (e-value < 1 × 10^−5^, percent match length ≥ 50%). Markov Cluster Algorithm (MCL) clustering was performed with an inflation parameter of 1.5. Ka/Ks ratios were calculated using PAML v4.8a [17] software with default settings.

### 2.4. Identification of Orthologous Groups

In the current study, two approaches were employed to determine genes associated with phototransduction and the circadian clock. First, candidate genes involved in phototransduction and the circadian clock for the three beetles were identified by conducting a keyword search of our BLAST annotation results. Second, TBtools II (v2.224) program was used and the sequences of *Drosophila melanogaster* and *Bombyx mori* were employed as queries to search the annotated protein sequences of *H. parallela*, *H. oblita*, or *A. corpulenta* with default parameters. For validation of core gene orthology, protein sequences from the *H*. *oblita* genome (GCA_023690525.1) were utilized.

For the purpose of comparing the expression values and determining the differentially expressed genes of orthology genes among three species, orthogroups that encompass orthologous candidate unigenes expressed in all the three species were chosen. For differential expression analysis, the read counts of all genes assigned to the orthogroups obtained from OrthoMCL (v2.0.3) clustering were extracted and analyzed using DESeq2 (v1.42.0). The Wald test was used for hypothesis testing, and p-values were adjusted for multiple comparisons using the Benjamini–Hochberg method. Orthogroups with |log_2_FC| > 1 and adjusted *p*-value (padj) < 0.05 were defined as significantly differentially expressed. This analysis follows the recommended strategy for multi-species transcriptomic comparisons [18]. To reduce potential host plant effects, genes that are typically influenced by diet, such as those involved in detoxification, digestion, and nutrient transport, were excluded from the main analysis. Bar plots of GO and KOG functional classifications and expression profiles were visualized using R software (v4.5.1). Protein–protein interaction analysis was carried out using the STRING database version 12 [19], with *Homo sapiens* and *D. melanogaster* as the query organisms, respectively.

## 3. Results

### 3.1. Sequencing and Quality Control

Adult head samples from males and females of *H. parallela*, *H. oblita*, and *A. corpulenta* were sequenced for transcriptome profiling. The transcriptomes of *H. parallela*, *H. oblita* and *A. corpulenta* were obtained by assembling the sequencing data of three biological replicates of male and female heads of each species separately. *H. parallela* produced 55,867,364 reads, which reduced to 52,254,625 after the quality control pipeline. The GC percentage was 35.53%, and the Q30 percentage was 95.98%. Sequencing of *H. oblita* produced 45,205,599 reads which reduced to 43,741,031 after quality control. The GC percentage was 37.32%, and the Q30 percentage was 95.89%. *A. corpulenta* produced 56,774,056 reads. After trimming, *A. corpulenta* libraries had 54,230,270 reads. The GC percentage was 39.07%, and the Q30 percentage was 97.54% (Table 1).

### 3.2. Transcriptome Assembly, Annotation and Functional Classification

We assembled transcriptomes for *H. parallela*, *H. oblita*, and *A. corpulenta*, yielding 105,051, 118,659, and 133,761 unigenes, respectively. The total lengths of the unigenes were 72,844,841 bp, 74,519,435 bp, and 89,122,625 bp, with mean lengths of 693 bp, 628 bp, and 666 bp, and N50 lengths of 1209 bp, 1021 bp, and 1164 bp, respectively. For *H. parallela*, 16.13% of the unigenes (16,945) were longer than 1000 bp, while for *H. oblita* and *A. corpulenta*, 14.22% (16,879) and 14.79% (19,786) of the unigenes, respectively, exceeded 1000 bp in length (Table 2).

A total of 32,343 unigenes (30.78% of all sequences in *H. parallela*), 39,480 unigenes (33.27% of all sequences in *H. oblita*), 53,163 unigenes (39.74% of all sequences in *A. corpulenta*) were annotated in at least one database (Table 3).

**Figure 1:**
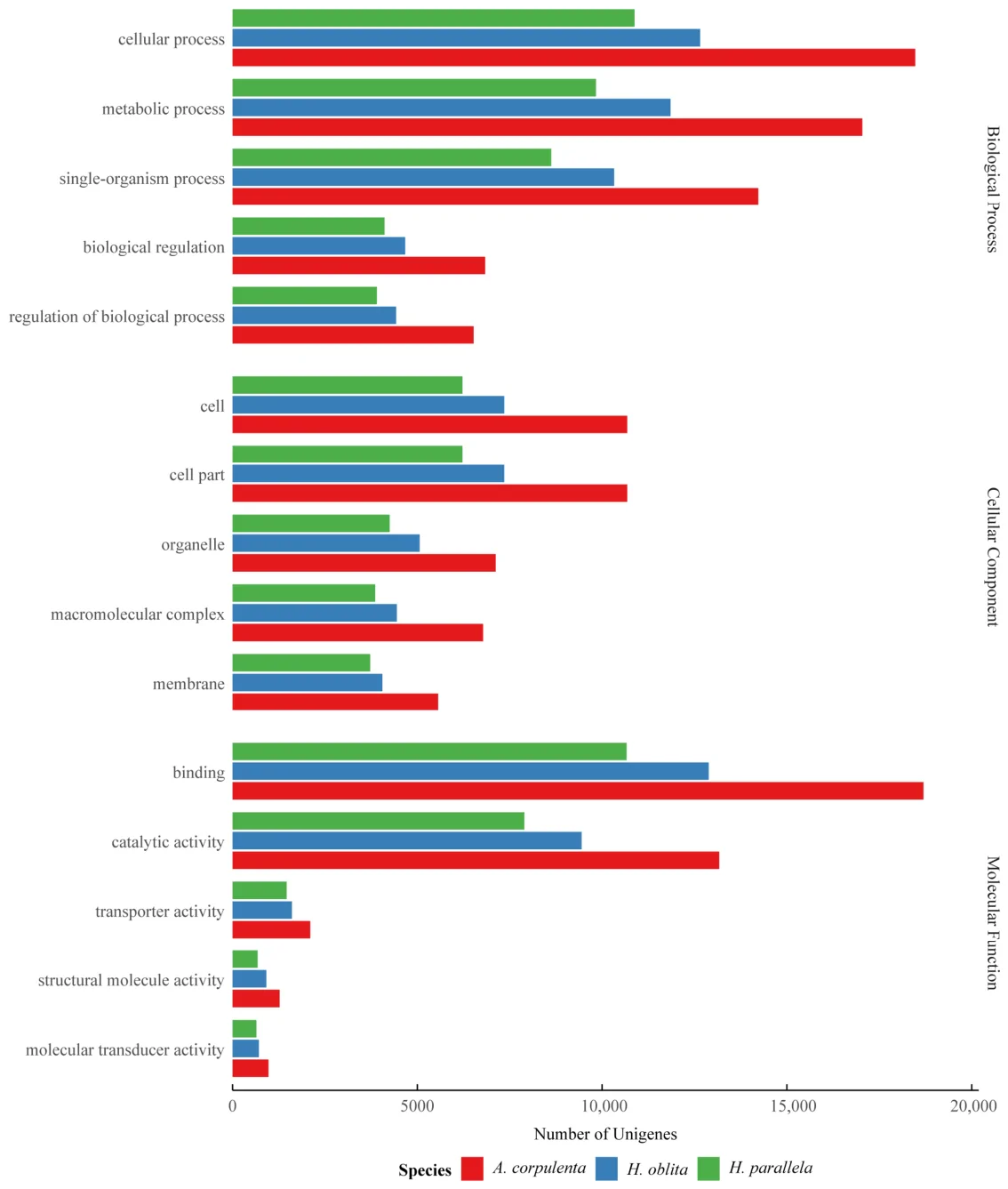
Distribution of gene ontology term categories within the transcriptomes of three beetle species.

For the three species, a BLASTX top-hit species distribution of gene annotations showed highest homology to *Tribolium castaneum* [10,481 of 21,970 hits in *H. parallela* (47.42%) vs. 12,825 of 30,442 in *H. oblita* (42.13%)] vs. 17,309 of 40,858 in *A. corpulenta* (42.36%), followed by *Dendroctonus ponderosae* (5.1% vs. 5.07% vs. 6.9%) (Supplementary Figure S1). The results showed that the two most suitable matches were the Coleoptera species of *T. castaneum* and *D. ponderosae*. Utilizing the GO classification system, we functionally categorized the unigenes based on NR annotations. A total of 19,827 (18.87%) unigenes for *H. parallela*, 23,680 (19.95%) unigenes for *H. oblita*, and 32,239 (24.84%) unigenes for *A. corpulenta* were assigned to at least one GO term annotation. The unigenes were assigned to three main GO categories: biological process (*H. parallela*: 50,771, 47.87%; *H. oblita*: 59,329, 47.86%; *A. corpulenta*: 85,344, 48.06%), cellular component (32,638, 30.77% vs. 37,571, 30.31%, vs. 53,701, 30.23%) and molecular function (22,661, 21.36% vs. 27,068, 21.83% vs. 38,540, 21.7%) (Figure 1). These GO terms were further subdivided into 54, 55, and 56 subcategories, respectively. For each species, the two largest subcategories of biological process were ‘cellular process’ and ‘metabolic process’; of cellular component, ‘cell’ and ‘cell part’; and of molecular function, ‘binding’ and ‘catalytic activity’. The gene ontology functional classification analysis revealed that the transcriptomes of the three beetle species are highly similar.

**Figure 2:**
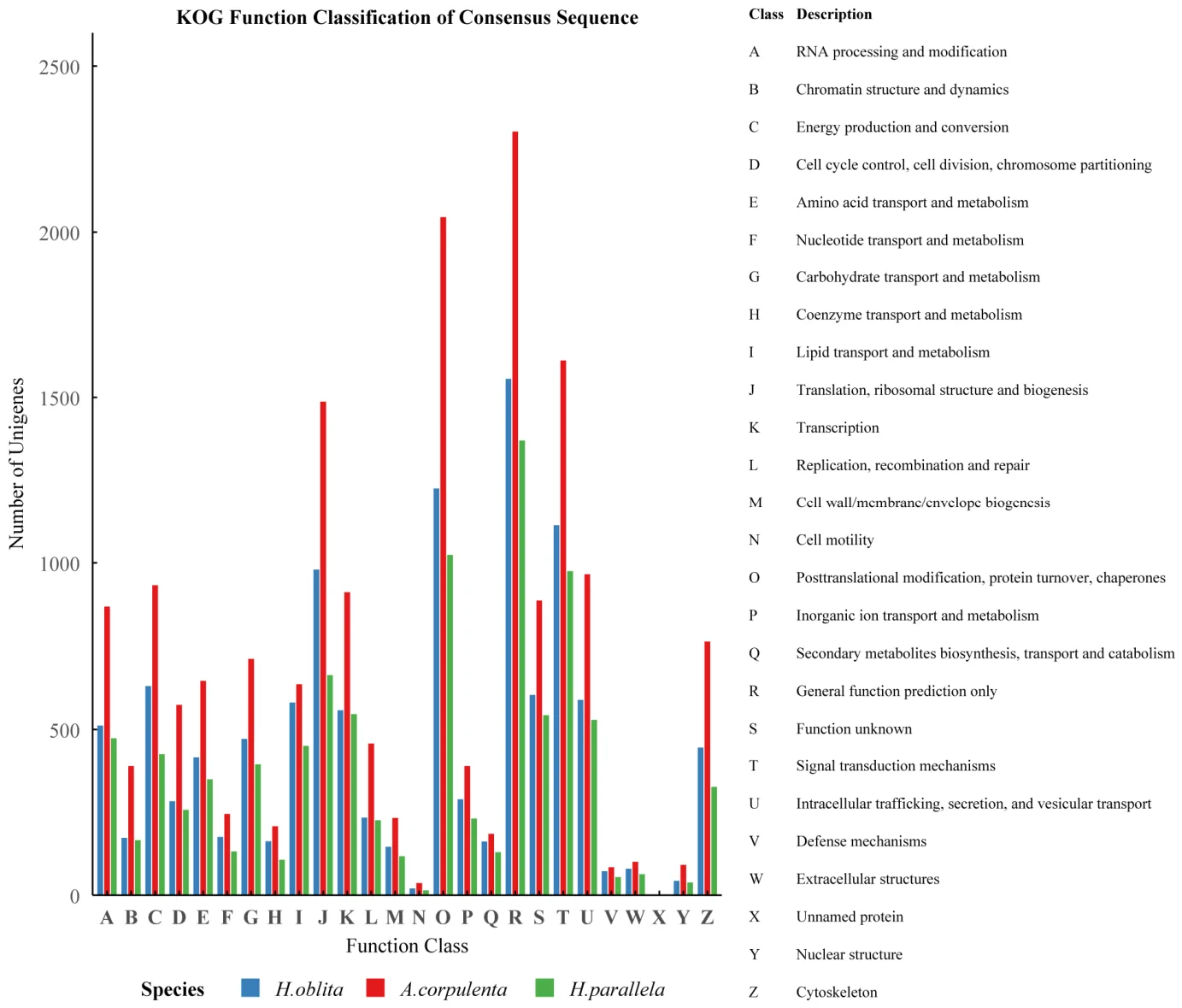
Functional classification of unigenes derived from the three transcriptomes is based on the KOG (eukaryotic Orthologous Groups) annotation.

To assess the completeness and efficacy of the annotation procedure, the unigenes that were matched with the NR database were subsequently classified using the KOG database, which is dedicated to the categorization of orthologous genes. A total of 8576unigenes for *H. parallela*, 10,405 unigenes for *H. oblita* and 15,978 unigenes for *A. corpulenta* were categorized into 25 KOG categories (A-W, Y and Z; Figure 2). The distributions of KOG classes were similar among the three transcriptomes. The biological function most commonly observed was [R] general function prediction only, with [O] posttranslational modification and [T] signal transduction mechanism following closely behind. In contrast, the least frequent category was [N] cell motility (Figure 2).

### 3.3. Protein-Coding Sequence (CDS) Prediction

Using BLASTX searches against protein database, we identified 17,569 unigenes with CDS regions in *H. parallela*, 19,989 in *H. oblita* and 28,217 in *A. corpulenta*. The distribution of sequence sizes among these unigenes varied as follows: 200–500 bp (6114/7503/12,155), 500–1000 bp (4025/4284/5961) and >1000 bp (6289/6506/7493) (Supplementary Table S1). Furthermore, utilizing ESTSCAN, we identified 12,829 unigenes containing CDS regions in *H. parallela*, 14,019 in *H. oblita*, and 20,783 in *A. corpulenta* that could not be aligned to any existing database. However, 94% of these unigenes were shorter than 500 bp in all three beetles.

### 3.4. Identification of Putative Orthologs and Substitution Rates in Three Beetles

**Figure 3:**
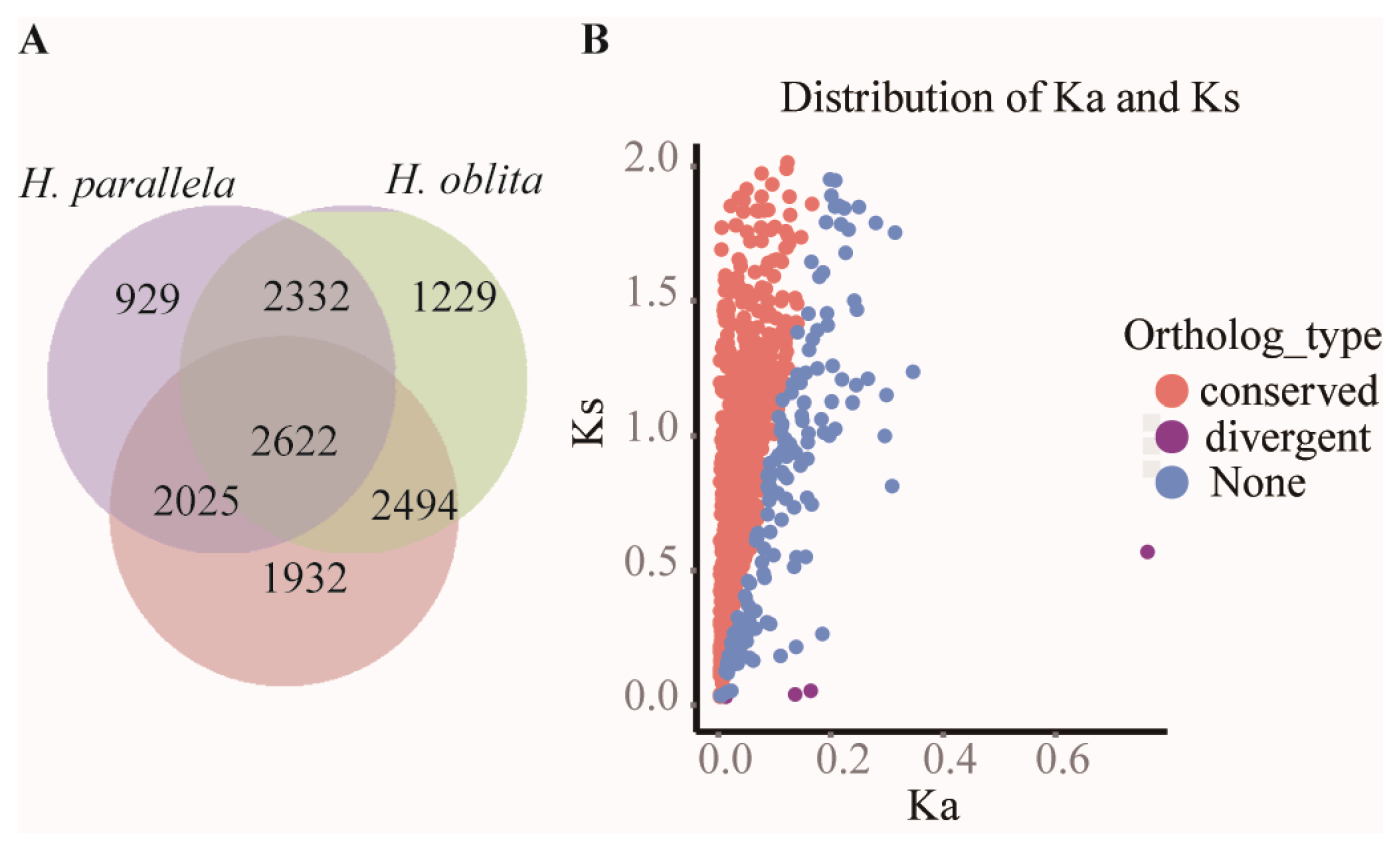
(**A**) Venn diagram presenting the number of specifics-specifics and overlapping orthologous protein groups among the three beetle transcriptome assemblies. Orthologous proteins are determined by OrthoMCL. (**B**) Scatter diagram of Ka and Ks values. Purple dots indicate Ka/Ks > 1, blue dots indicate 0.1 ≤ Ka/Ks ≤ 1, and red dots indicate Ka/Ks < 0.1.

After finalizing the annotations for the three beetle species under examination, 2019 genes were retained as highly reliable orthologous gene pairs. Using the OrthoMCL approach, a total of 2622 paired orthologues were detected among the three transcriptomes and with 404 orthologues compared with *T. castaneum* and *B. mori* (Figure 3A).

For the classification of genes undergoing purifying and positive selections, the substitution rates at synonymous (Ks) and non-synonymous (Ka) sites were determined. Excluding the sites with either synonymous or non-synonymous substitutions, 1313 pairs had both types of substitutions, thereby enabling the calculation of Ka/Ks ratios. Among the 1313 orthologues, only five pairs exhibited Ka/Ks ratios greater than 1, indicating these genes may be under strong positive selection and thus represent divergent orthologues. Two of the genes are related to afadin and venom protease-like proteins, while the others are uncharacterized. Additionally, eight orthologue pairs showing Ka/Ks = 1 underwent neutral selection; whereas, 1127 pairs displaying Ka/Ks < 0.1 exhibited strong purifying selection, thus classified as conserved orthologues (Figure 3B, Supplementary Table S2).

### 3.5. Candidate Genes Involved in Phototransduction and Circadian Clock

**Figure 4:**
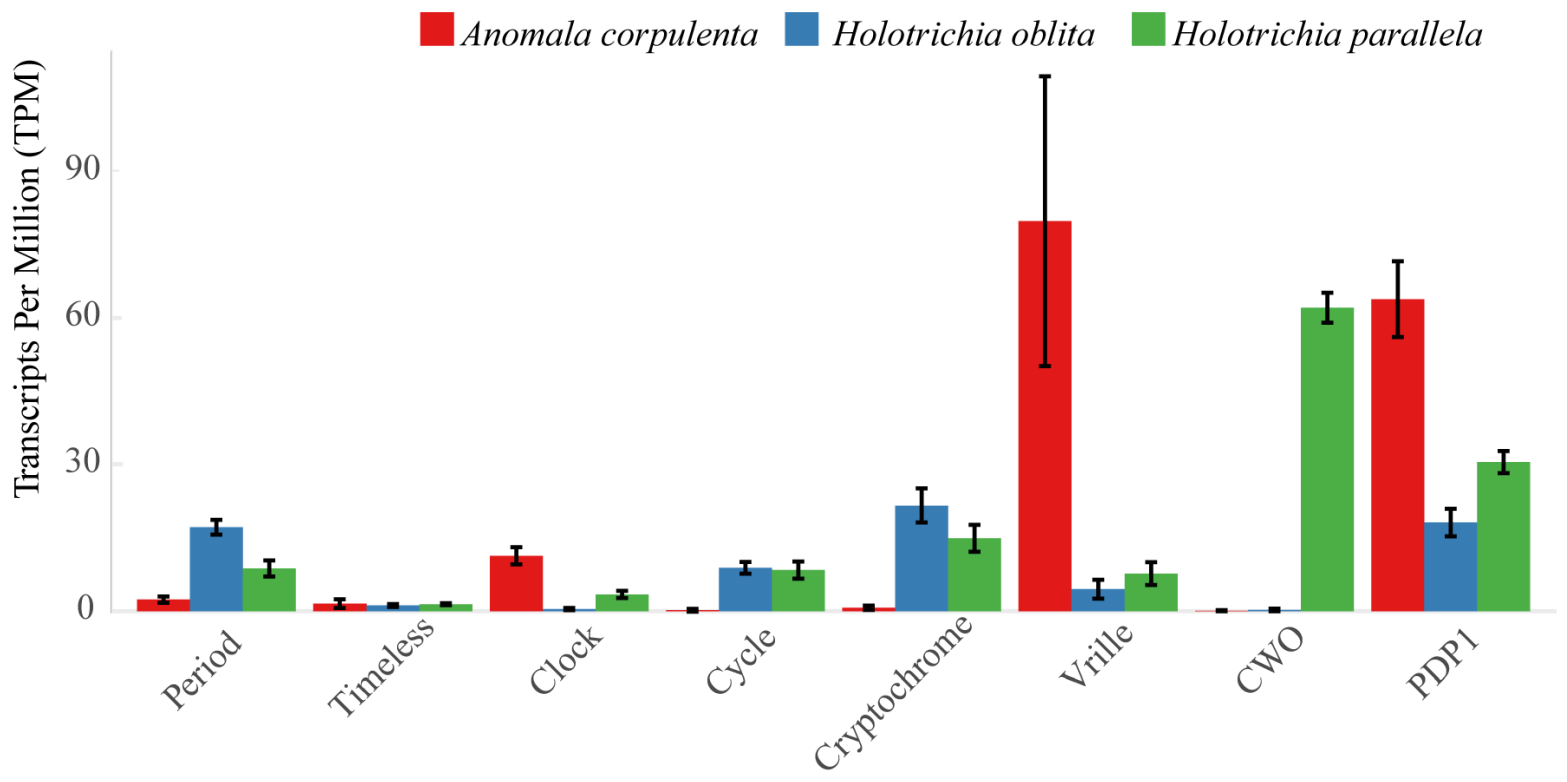
Transcript abundances of core circadian clock genes at 22 h. Bar plot showing raw TPM values of eight circadian clock genes in three scarab beetle species: *Anomala corpulenta* (red), *Holotrichia oblita* (blue), and *Holotrichia parallela* (green). Data were collected from head tissues at 22:00 h. Values represent mean TPM ± SD of six biological replicates (combining HF and HM, *n* = 3 each). Individual TPM values for each replicate are provided in Table S3. Gene abbreviations: CWO = Clockwork orange, PDP1 = PAR-domain protein 1.

Among gene products known to relate to circadian clock genes, the canonical clock genes are the beststudied examples. While many circadian genes have been characterized in various insects, only the pigment-dispersing factor (pdf) gene has been reported to potentially contribute to the circabidian rhythm in *H. parallela* [20]. Expression analysis of core circadian genes (e.g., *per*, *tim*, *cry*, *clk*, *cyc*) revealed no consistent expression pattern that clearly distinguishes the circabidian rhythm of *H. parallela* from the circadian rhythms of the other two species at the sampled time point, based on TPM values at 22:00 h. The TPM values of the eight circadian clock genes at 22:00 h varied among the three species (Figure 4, Supplementary Table S3). In *A. corpulenta*, *vrille* (79.81 ± 27.09 TPM) and *pdp1* (63.82 ± 7.09 TPM) showed the highest expression. cwo (0.07 ± 0.10), cycle (0.19 ± 0.27), and cryptochrome (0.70 ± 0.39) were expressed at low levels. In *H. oblita*, *cryptochrome* (21.62 ± 3.16), *period* (17.20 ± 1.37), and *pdp1* (18.15 ± 2.56) were the most highly expressed transcripts; whereas, *clock* was nearly undetectable (0.43 ± 0.21). *H. parallela* exhibited a distinct pattern, with *cwo* (62.08 ± 2.80) and *pdp1* (30.46 ± 2.06) showing the highest expression. *Timeless* showed comparable TPM values across all three species (1.53 ± 0.81, 1.17 ± 0.25, and 1.42 ± 0.19, respectively).

**Figure 5:**
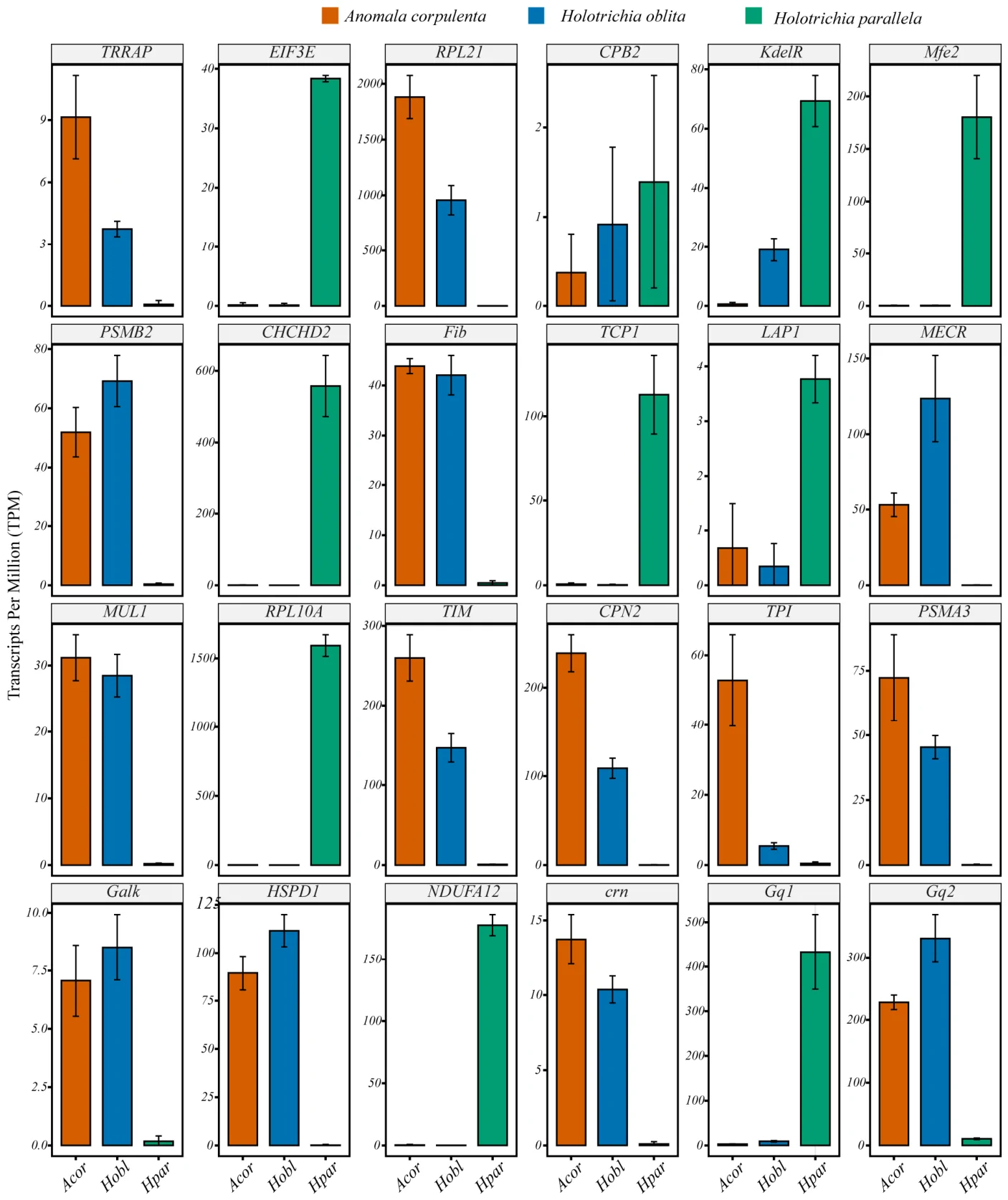
Transcripts per million (TPM) expression values for selected genes at 22:00 h. Faceted bar plot showing raw TPM values of 24 selected genes in three scarab beetle species: *Anomala corpulenta* (orange), *Holotrichia oblita* (blue), and *Holotrichia parallela* (green). Data were collected from head tissues at 22:00 h. Each panel represents an individual gene with its own *Y*-axis scale to accommodate the wide range of expression levels across different genes. Values represent mean TPM ± SD of six biological replicates (HF and HM, *n* = 3 each). Individual TPM values for each replicate are provided in Table S4.

To identify candidate genes that may be associated with species-specific behavioral traits, we screened for genes with expression bias in *H. parallela* relative to the other two species at a single time point (22:00 h). Among the genes examined, several are involved in protein synthesis, including *EIF3E*, *RPL21* and *RPL10A*. Genes associated with ribosome synthesis and transcriptional regulation, including TRRAP, were observed. Among the 24 genes examined, six showed markedly higher expression in *H. parallela* than in the other two species (Figure 5). Notably, six genes (*RPL10A*, *CHCHD2*, *Mfe2*, *NDUFA12*, *TCP1*, and *EIF3E*) showed markedly higher expression in *H. parallela* compared to the other two species. Among these, *RPL10A* exhibited the most striking difference, with TPM values of 1593.66 in *H. parallela*, compared to only 1.65 in *A. corpulenta* and 0.72 in *H. oblita*. Similarly, *CHCHD2* (557.91 TPM), *Mfe2* (180.33), *NDUFA12* (177.66), *TCP1* (112.74), and *EIF3E* (38.35) were all highly expressed in *H. parallela* but were present at very low levels (<5 TPM) in both *A. corpulenta* and *H. oblita*. These results showed TPM values in *H. parallela* for genes involved in mitochondrial function (*CHCHD2*, *NDUFA12*), lipid metabolism (Mfe2), protein folding (*TCP1*), and translation (*RPL10A*, *EIF3E*). In contrast, *RPL21*, *PSMB2*, *Fib*, *MECR*, *MUL1*, *TIM*, *CPN2*, *TPI*, *PSMA3*, *Galk*, *HSPD1* and *crn* showed higher observed expression in *H. oblita* and *A. corpulenta* than in *H. parallela* (Figure 5, Supplementary Table S4). No significant sex-biased differences were observed for any of these genes.

*PSMA3* and *PSMB2*, which showed higher expression in *H. oblita* and *A. corpulenta*, encode components of the ubiquitin-proteasome system and are involved in protein degradation and ubiquitination.

Gq, a type of heterotrimeric protein, is responsible for generating two second messengers, inositol trisphospate (IP3) and diacylglycerol (DAG), both of which are involved in mobilizing calcium from intracellular storage to initiate cellular events [21]. At the 22:00 h time point, we observed TPM values for Gq1 and Gq2 in *H. parallela*, *H. oblita*, and *A. corpulenta* (Figure 5). For Gq1, the observed TPM values were 433.34 in *H. parallela*, 9.30 in *H. oblita*, and 3.05 in *A. corpulenta*. For Gq2, the observed values were 10.60 in *H. parallela*, 330.64 in *H. oblita*, and 228.03 in *A. corpulenta* (Figure 5).

Taken together, genes involved in protein synthesis and protein degradation showed differential expression patterns among the three species at the 22:00 h collection time point. At the 22:00 h time point, we observed TPM values for these genes in all three species. The observed values for individual genes varied across species (Table S4).

**Figure 6:**
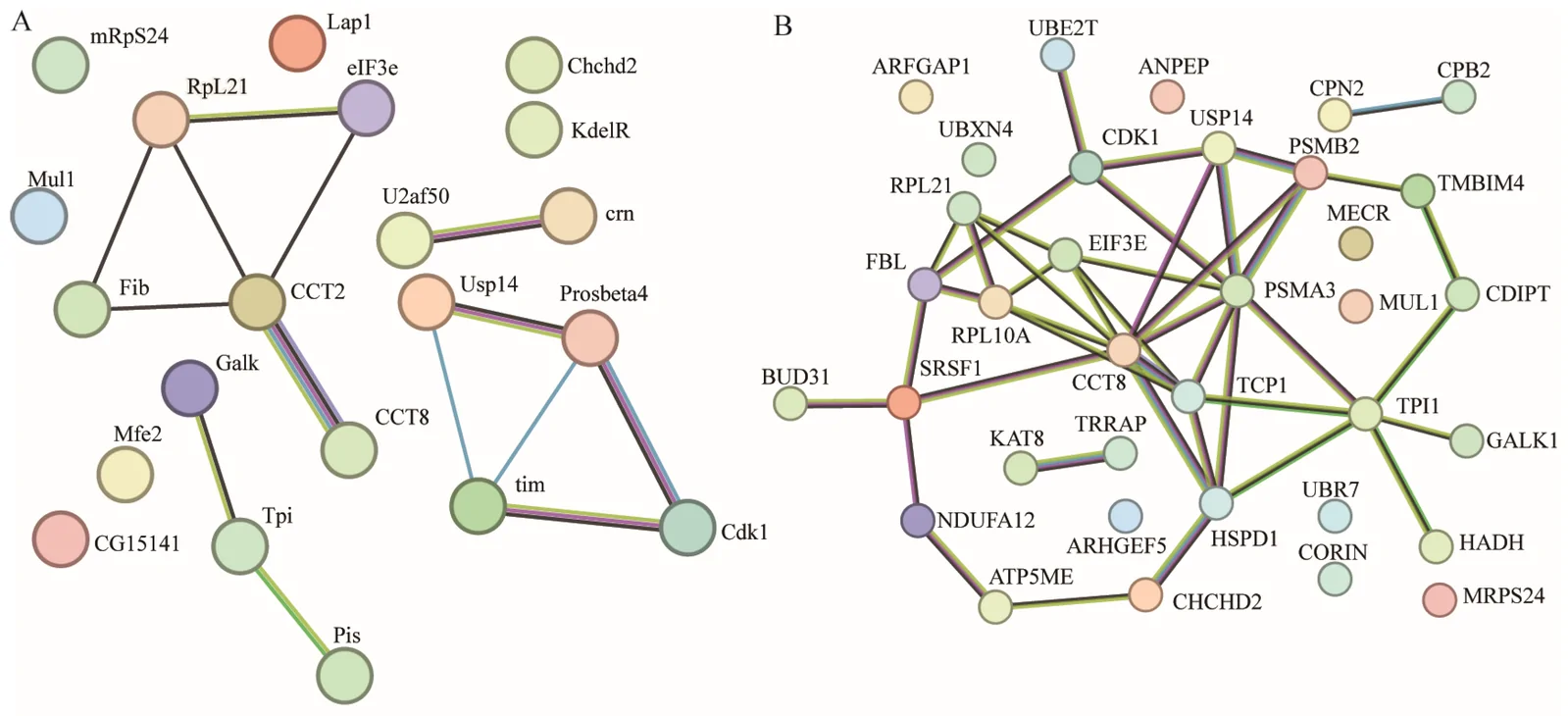
Complete protein–protein interaction network of the identified orthology genes. The network was determined by uploading the gene list into STRING. (**A**) *D. melanogaster* as the query organisms. (**B**) *Homo sapiens* as the query organism. Colored lines denote the types of interaction evidence between proteins.

Using STRING-based cross-species projection, we identified potential interactions among several of the selected proteins, including RPL21 and EIF3E (Figure 6). When *Drosophila* was used as a reference, certain genes lacked annotation in the *Drosophila* dataset (Figure 6A); whereas, the *Homo sapiens* protein interaction data were more comprehensive (Figure 6B). Therefore, we used the *Homo sapiens* database to generate a predicted interaction network for exploratory visualization.

To account for evolutionary distance, we compared both datasets and focused on interactions that were commonly predicted in both species. These shared predictions suggest that the interacting proteins may be evolutionarily conserved. However, we noted that these predicted interactions are bioinformatic inferences. Further experimental validation, such Co-IP and yeast two-hybrid assays is needed to confirm their presence in scarab beetles. Thus, while these predicted interactions can serve as a hypothesis-generating resource for future functional studies, they remain highly inferential and are not conclusive evidence of protein–protein interactions in *H. parallela*.

## 4. Discussion

In the present study, we performed de novo transcriptome sequencing and assembly on head tissues of male and female *H. parallela*, *H. oblita*, and *A. corpulenta* collected at a single time point (22:00 h). This work provides a descriptive transcriptome resource for these three scarab beetle species. We identified orthologs and compared expression levels across the three species at this time point. Core circadian clock genes showed comparable expression levels across species, and no sex-biased expression were observed for these genes at the sampled time point. Differences in expression were observed in phototransduction pathway genes (Gq1/Gq2), and 22 other orthologous genes, including those involved in protein synthesis and the ubiquitin-proteasome system.

Importantly, because sampling was limited to a single time point and the species were collected under different behavioral states, the expression differences reported here may reflect behavioral state rather than rhythm of locomotor activity. At the sampled time point, we observed expression patterns in *H. parallela* for the genes examined, providing a foundation for future studies.

Orthologues of core circadian clock genes (*per*, *timeless*, *cry2*, *pdp1*, *clk*, *cwo*, *vri*, and *cyc*) were detected in *H. parallela*, and TPM values for these genes were also observed in *H. oblita* and *A. corpulenta* at the 22:00 h time point (Figure 4). No sex-biased expression was observed for these genes in any of the three species at this time point.

This preliminary observation from a single time point does not rule out the involvement of the circadian clock in rhythm of locomotor activity. However, it provides a descriptive reference for future studies that may investigate whether the 48h interval rhythm, as suggested for *Platynereis dumerilii* [22], could involve a separate molecular oscillator.

In invertebrates, phototransduction has been most thoroughly studied in *Drosophila*, where Gq/11-type G-proteins play a central role [23,24,25]. In the present study, the unigene annotated as Gq1 showed higher expression in *H. parallela* than in *H. oblita* and *A. corpulenta*; whereas, Gq2 showed lower expression in *H. parallela* than in the other two species.

In *Drosophila*, the *dgq* gene undergoes alternative splicing, leading to the production of two distinct variants of the Gqα protein [26]. Both electrophysiological and genetic studies provide strong evidence that DGq1 and DGq2 exert unique functions within photoreceptor cells [27,28]. Seven Gαq splice variants have been annotated in flyBase, with several distinct Gq isoform proteins playing different roles in phototransduction [29].

Physiological studies showed that the axis between the optic lobe and the pars intercerebralis was involved in circabidian rhythm in *H. parallela*. The circabidian rhythm vanished following the bilateral removal of the optic lobe (OL). Additionally, the partial removal of the PI cells led to arrhythmicity in some individuals [9]. The observed expression differences in Gq1 and Gq2 in the present study are consistent with the possibility that these genes may be relevant to visual or circadian functions in *H. parallela*. However, causal conclusions cannot be drawn from the current data.

PSMB2 and PSMA3 are components of the ubiquitin-proteasome system (UPS), which is involved in protein degradation and contributes to circadian clock regulation through the degradation of core clock proteins in other species [30]. In the present study, *PSMB2* and *PSMA3* expression levels were lower in *H. parallela* than in *H. oblita* and *A. corpulenta*.

Ubiquitin is attached to target proteins by three enzymes: the ubiquitin-activating enzyme E1 (MUL1), the ubiquitin-conjugating enzyme E2 (UBE2T), and the ubiquitin ligase E3 (UBR7) [31,32]. In this study, *MUL1* and *UBR7* showed lower expression in *H. parallela*; whereas, *UBE2T* showed higher expression in *H. parallela* compared to the other two species. These contrasting patterns indicate that different components of the UPS show distinct expression profiles across species at the sampled time point.

The UPS is involved in diverse biological functions, including responses to environmental stimuli and regulation of circadian rhythms in other species [30,31,33]. UBE2T, together with tumor necrosis factor receptor-associated factor 7 (TRAF7, a RING-type E3 ligase), facilitates the degradation of D-site binding protein (DBP). TRAF7 determines circadian period through ubiquitination [33]. However, the functional relevance of the observed expression differences in *H. parallela* remains unknown.

Distinct expression patterns of orthologous genes were observed among the three scarab beetle species based on TPM values at the sampled time point, with each species showing a different profile. Notably, *HparOR14* was not annotated in our head transcriptome assemblies. This is consistent with its predominant expression in antennae rather than head tissue [7]. It was therefore excluded from our differential expression analysis. Due to the single-time-point design and the different behavioral states at collection (active vs. non-active), the observed expression differences cannot be causally attributed to rhythm of locomotor activity. These data provide a set of candidate genes and pathways for future studies on behavioral rhythm divergence in Coleoptera. Additional transcriptomic sampling across multiple time points from all three species will be required to further explore the relationship between these expression differences and rhythm of locomotor activity.

## 5. Conclusions

This study provides a descriptive head transcriptome resource for three scarab beetle species collected at 22:00 h. We observed expression differences in phototransduction, mitochondrial metabolism and ubiquitin-proteasome pathway genes based on TPM values at this time point. Core circadian clock genes showed comparable TPM values at this time point. These data offer a candidate gene set and a descriptive foundation for future studies on behavioral rhythm divergence in closely related beetles.

## Figures and Tables

**Table 1:** Summary of Illumina short read sequencing for the three beetles, *Holotrichia parallela*, *Holotrichia oblita* and *Anomala corpulenta*.

Library	Tissue	Sequencing Technology	Raw Reads	Clean Reads	GC Content (%)	Q20 (%)	Q30 (%)
*H. oblita*	Head	HiSeq	45,205,599	43,741,031	37.32	95.89	89.85
*H. parallela*	Head	HiSeq	55,867,364	52,254,625	35.53	95.98	91.15
*A. corpulenta*	Head	HiSeq	56,774,056	54,230,270	39.07	97.54	93.98

**Table 2:** The assembly results of the transcriptomes of the three beetles, *Holotrichia parallela, Holotrichia oblita* and *Anomala corpulenta*. The numbers of unigenes in different length intervals are provided along with the percentage of all identified unigenes (in parentheses).

Length Intervals	*Anomala corpulenta*	*Holotrichia oblita*	*Holotrichia parallela*
200–500 bp	93,207 (69.68%)	83,464 (70.34%)	70,579 (67.19%)
500–1 k bp	20,768 (15.53%)	18,316 (15.44%)	17,527 (16.68%)
1 k–2 k bp	11,130 (8.32%)	10,141 (8.55%)	9622 (9.16%)
>2 K bp	8656 (6.47%)	6738 (5.68%)	7323 (6.97%)
Total number of unigenes	133,761	118,659	105,051
Total length of unigenes	89,122,625	74,519,435	72,844,841
N50 length of unigenes	1164	1021	1209
Mean length of unigenes	666	628	693

**Table 3:** The annotation results of the transcriptomes of the three beetles, *Holotrichia parallela, Holotrichia oblita* and *Anomala corpulenta*.

Database	*Anomala corpulenta*		*Holotrichia oblita*		*Holotrichia parallela*	
	Number	Percent (%)	Number	Percent (%)	Number	Percent (%)
Annotated in NR	40,858	30.54	30,442	25.65	21,970	20.91
Annotated in NT	12,723	9.51	10,427	8.78	11,347	10.8
Annotated in KO	17,242	12.89	11,525	9.71	7306	6.95
Annotated in SwissProt	29,769	22.25	19,586	16.5	16,403	15.61
Annotated in PFAM	32,111	24	23,181	19.53	19,544	18.6
Annotated in GO	33,239	24.84	23,680	19.95	19,827	18.87
Annotated in KOG	15,978	11.94	10,405	8.76	8576	8.16
Annotated in all Databases	3488	2.6	3173	2.67	2318	2.2
Annotated in at least one Database	53,163	39.74	39,480	33.27	32,343	30.78
Total Unigenes	133,761	100	118,659	100	105,051	100

## Data Availability

The raw read count matrix and TPM expression matrix generated in this study are available as Supplementary Materials (Tables S5 and S6). The raw sequencing data reported in this paper have been deposited at NGDC (National Genomics Data Center, Beijing Institute of Genomics, Chinese Academy of Sciences) [34] under the accession number CRA011607 and CRA011598.

## Supplementary Materials

The following supporting information can be downloaded at: https://www.mdpi.com/article/10.3390/insects17080781/s1. Figure S1: Nr Homologous Species Distribution. Species distribution is shown as a percentage of the total homologous sequences with an e-value of at least 1.0 × 10^−5^ in *H. parallela* all-transcripts (A), *H. oblita* all-transcripts (B) and *A. corpulenta* all-transcripts (C). We used the first hit of each sequence for analysis; Table S1: Unigene.blast.cds.length statistics; Table S2: Ka/Ks values; Table S3: Clock_genes_TPM; Table S4: Orthogroup_genes_TPM; Table S5: ReadCount_matrix_all_samples; Table S6: TPM_expression_matrix_all_samples.
