# Supplementary material for: A Descriptive Transcriptomic Resource of Head Tissues from Three Scarab Beetle Species at a Single Time Point Corresponding to Divergent Behavioral States
Source: Insects. 2026 Jul 28;17(8):781. doi: 10.3390/insects17080781 (PMC13513377; doi:10.3390/insects17080781)
Supplement: Supplementary file 1 [file insects-17-00781-s001.zip › Supplementary Figure.pdf]

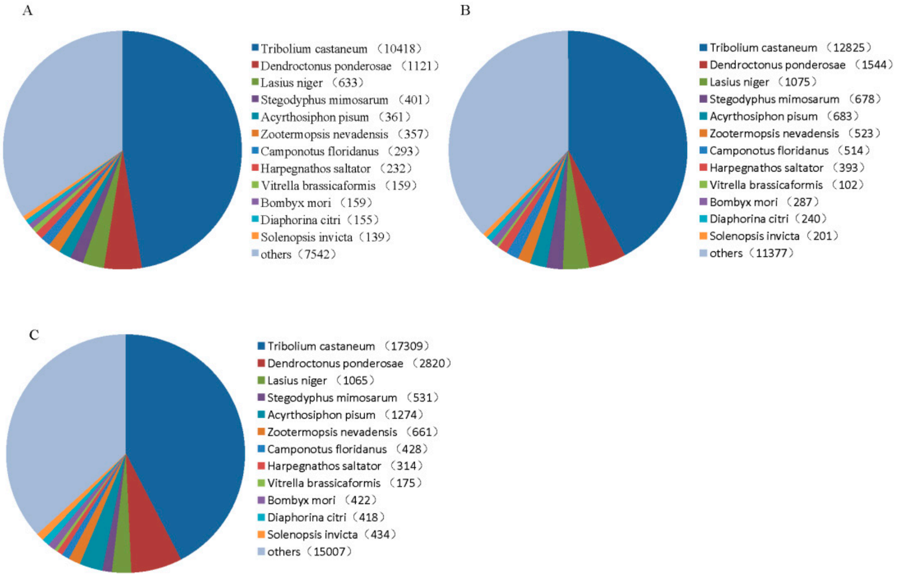

Figure S1 Nr Homologous Species Distribution

Species distribution is shown as a percentage of the total homologous sequences with an E-value of at least 1.0E^{-5} in *H. parallela* all- transcripts (A), *H. oblita* all- transcripts (B) and *A. corpulenta* all-transcripts (C). We used the first hit of each sequence for analysis.
